# Structural Proteomics of Herpesviruses

**DOI:** 10.3390/v8020050

**Published:** 2016-02-12

**Authors:** Baptiste Leroy, Laurent Gillet, Alain Vanderplasschen, Ruddy Wattiez

**Affiliations:** 1 Laboratory of Proteomic and Microbiology, Research Institute of Biosciences, University of MONS, 4000 Mons, Belgium; ruddy.wattiez@umons.ac.be; 2 Immunology-Vaccinology, Department of Infectious and Parasitic Diseases, Fundamental and Applied Research for Animals and Health, Faculty of Veterinary Medicine, University of Liege, 4000 Liege, Belgium; l.gillet@ulg.ac.be (L.G.); a.vdplasschen@ulg.ac.be (A.V.)

**Keywords:** herpesvirus, structural proteins, proteomic, host proteins

## Abstract

Herpesviruses are highly prevalent viruses associated with numerous pathologies both in animal and human populations. Until now, most of the strategies used to prevent or to cure these infections have been unsuccessful because these viruses have developed numerous immune evasion mechanisms. Therefore, a better understanding of their complex lifecycle is needed. In particular, while the genome of numerous herpesviruses has been sequenced, the exact composition of virions remains unknown for most of them. Mass spectrometry has recently emerged as a central method and has permitted fundamental discoveries in virology. Here, we review mass spectrometry-based approaches that have recently allowed a better understanding of the composition of the herpesvirus virion. In particular, we describe strategies commonly used for proper sample preparation and fractionation to allow protein localization inside the particle but also to avoid contamination by nonstructural proteins. A collection of other important data regarding post-translational modifications or the relative abundance of structural proteins is also described. This review also discusses the poorly studied importance of host proteins in herpesvirus structural proteins and the necessity to develop a quantitative workflow to better understand the dynamics of the structural proteome. In the future, we hope that this collaborative effort will assist in the development of new strategies to fight these infections.

## 1. Introduction

The order *Herpesvirales* contains a large number of viruses that share genetic, structural, and biological properties. It is divided into three phylogenetically related families infecting a wide range of hosts [1]. The *Malacoherpesviridae* family comprises viruses that infect molluscs. The *Alloherpesviridae* family encompasses viruses that infect fish and amphibians. Finally, the *Herpesviridae* family encompasses viruses that infect mammals, birds, or reptiles and it is by far the most important, both in terms of the number of its members and the volume of studies that have been devoted to them. The *Herpesviridae* family is subdivided into three subfamilies, the *Alpha*-, *Beta*-, and *Gamma-herpesvirinae* [2]. Members of these subfamilies are referred to colloquially as alpha-, beta-, and gamma-herpesviruses.

Proteomics is a highly valuable tool for studying virus structure and understanding mechanisms by which viruses can replicate and spread using the host cellular machinery. Frequently, the cellular response to virus infection is analyzed by looking at changes in cellular protein abundances and post-translational modifications but also viral-host protein interactions (for a global review see [3,4]). The contribution of proteomic analyses to the understanding of the life cycle of alphaherpesviruses have been recently reviewed by Engel *et al.* [5]. In addition to understanding the host response to viral infection, a description of the particle composition both in terms of viral and cellular proteins is also of major importance for a better characterization of infectious processes. In particular, characterization of the composition of complex enveloped viruses, such as poxviruses and herpesviruses, is of major importance to better understand their life cycle. Knowledge of the composition of the virion but also of the distribution of proteins in the capsid, tegument, and envelope are essential to understand virion assembly [6] or initiate functional characterization of structural proteins through targeted approaches such as interactomics [7]. Davison and Davison [8] published the first report of the use of mass spectrometry for determination of the structural proteome of a herpesvirus, Ictalurid herpesvirus 1 (IcHV-1), which is also known as channel catfish virus. Several other studies have since been performed to characterize the structural proteins of the virion of herpesviruses. It has been demonstrated that knowledge of the composition of virions can be highly useful for the development of improved vaccines and diagnostic tools for the control of herpesvirus infections [9].

This review summarizes the different aspects of the study of the structural proteome focusing on the *Herpesviridae* family, notably the different proteomic workflow that has been successfully used and the importance of sample preparation. The review also discusses the status of host cellular proteins found in the virion and the interest in acquiring quantitative data.

## 2. Mass Spectrometry Based Proteomics

**Figure 1:**
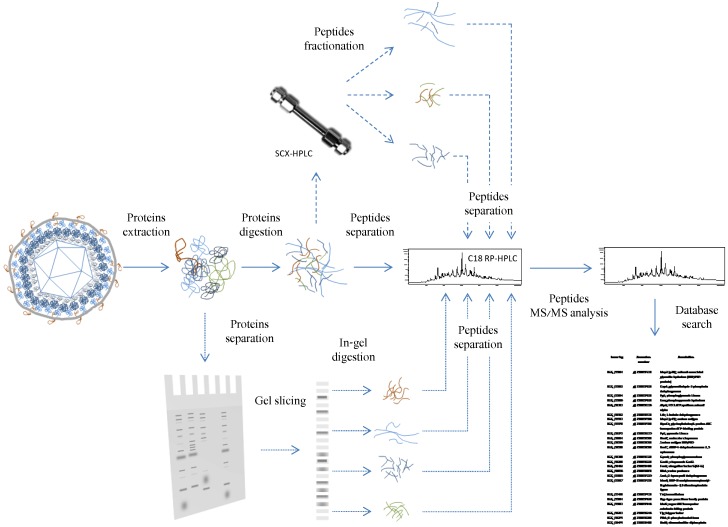
Common proteomic workflows that are applied for determination of the structural proteome. In shotgun proteomic analysis (solid lines), the proteins are extracted, digested in solution and derived peptides are separated according to their hydrophobicity using reversed phase reverse phase-high-performance liquid chromatography (RP-HPLC) and submitted to tandem mass spectrometry (MS/MS) analysis. Alternatively, proteome coverage can be enhanced by protein separation using sodium dodecyl sulfate polyacrylamide gel electrophoresis (SDS-PAGE) (dotted lines). Enzymatic digestion in this case takes place “in-gel” and eluted peptides are then submitted to liquid chromatography (LC)-MS/MS as in the shotgun analysis. This type of strategy is efficient in reducing the impact of the high dynamic range of the concentration. In Multi-Dimensional Protein Identification Technology (MuDPIT) approaches (dashed lines), protein digestion takes place in solution as in shotgun analyses but the peptide separation is performed using two-dimensional chromatography. Peptides are separated according to their charge using strong cation exchange (SCX) chromatography followed by the usual reverse phase chromatography as in the shotgun analysis. The number of fractions obtained from SCX chromatography needs to be adapted based on the sample complexity and usually ranges from 3 to 10.

In a classical modern mass spectrometry (MS) based proteomic workflow, proteins are digested with a specific enzyme (trypsin being the most commonly used) to produce peptides. Those peptides are separated by reverse-phase liquid chromatography and on-line infused in the mass spectrometer through the electrospray ionization (ESI) source (Figure 1, solid lines). The mass spectrometer acquires survey spectra from which peptides to be fragmented are selected. The main limitation of this type of workflow, called shotgun proteomics, comes from the co-elution of too many peptides for the mass spectrometer to address and from the presence of very abundant peptides, which impair detection of the ones that are less abundant. These two effects are derived from the sample complexity and the dynamic range of protein concentration. Different strategies have been proposed to circumvent these issues and improve the efficiency of the approach [10,11]. Sodium dodecyl sulfate polyacrylamide gel electrophoresis (SDS-PAGE) fractionation followed by in-gel trypsin digestion of proteins and liquid chromatography-tandem mass spectrometry (LC MS/MS) analysis of the peptides produced is one of the most commonly used strategies (Figure 1, dotted lines). Alternatively, proteins can be digested in solution and the peptides further separated following two orthogonal chromatography (Figure 1, dashed lines) before analysis in MS, and this method is called two-dimensional online liquid chromatography-mass spectrometry (2D-LC-MS/MS) or Multi-Dimensional Protein Identification Technology (MuDPIT). In the context of the characterization of the structural proteome of viruses, the sample complexity is rather low with generally less than 100 proteins being expected (virus + host proteins), whereas the dynamic range of the protein concentration can be very high with some capsid and tegument proteins being a thousand time more abundant than other structural proteins [12]. Different workflows were used for identification of the herpesvirus structural proteins. Table 1 shows that gel based separation of the proteins followed by LC MS/MS analysis of the peptides is by far the most often used strategy. The rational for selecting gel-based approaches rather than gel-free methods are numerous. First, SDS-PAGE separation of protein is a very simple procedure, which is accessible in almost every lab. Second, SDS-PAGE fractionation of the sample allows one to decrease the detrimental effect of the high dynamic range of the protein concentration in herpesvirus samples. Indeed, fractionation at the protein level (rather than the peptide level in gel-free approaches) decreases the number of fractions in which the proteins with the highest concentration can impair detection of the protein with the lowest concentration. For example, the major capsid protein, which is a very abundant protein, will only be detected in some regions of the gel and will not impair detection of proteins migrating in the other region of the gel. However, this advantage of the gel-based approach should be relativized because many proteins in herpesvirus samples appear as multimeric entities and/or isoforms and consequently do not migrate at a single location in the gel. Thorough denaturation of proteins (using sufficient heating in the sample preparation step) and deglycosylation of the proteins can sometimes help decrease the spreading of the proteins throughout the gel lane. A third rational for using gel-based approaches is the higher capacity of sample loading. A total of 10–30 µg of proteins are commonly loaded on a SDS-PAGE gel (compare to 2–5 µg in gel-free approaches), which increases the chance of detecting low abundance proteins. When the sample availability is not an issue, this high loading capacity is definitely an asset. It is important here to point out the difference between the targeted and untargeted strategy in gel-based protein identification workflows as mentioned in Table 1.

**Table 1:** Strategies used in the analyses of the structural proteome of herpesviruses.

Authors	Year of Publication	Virus	Separation Used	Targeted or Untargeted Identification	Mass Spec. Strategy
Davison et Davison [8]	1995	CCV	Gel based	Targeted	Peptide mass fingerprint
Nealon *et al.* [13]	2001	KSHV	Gel based	Targeted	LC MS/MS
Bortz *et al.* [14]	2003	MHV6	Gel based	Targeted	LC MS/MS
Varnum *et al.* [15]	2004	HCMV	Gel free (2D)	Untargeted	LC MS/MS
Johannsen *et al.* [16]	2004	EBV	Gel based	Untargeted	LC MS/MS
Kattenhorn *et al.* [17]	2004	MCMV	Gel based	Targeted	LC MS/MS
Bechtel *et al.* [18]	2005	KSHV	Gel based	Targeted	MS/MS (Maldi Tof/Tof)
Zhu *et al.* [19]	2005	KSHV	Gel based	Targeted	LC MS/MS
O’connor *et al.* [20]	2006	RRV	Gel free + Gel based	Untargeted	LC MS/MS
Michael *et al.* [21]	2006	PRV	Gel based (1D + 2D)	Targeted	Peptide mass fingerprint
Loret *et al.* [22]	2008	HSV-1	Gel based	Targeted	LC MS/MS
Dry *et al.* [23]	2008	AlHV1	Gel based	Untargeted	LC MS/MS
Kunec *et al.* [24]	2009	CCV	Gel free (2D)	Untargeted	LC MS/MS
Michel *et al.* [25]	2010	CyHV-3	Gel based + Gel free (2D)	Untargeted	LC MS/MS
Kramer *et al.* [26]	2011	PRV	Gel based + Gel free	Untargeted	Maldi + ESI MS/MS
Van Beurden *et al.* [27]	2011	AngHV-1	Gel based + Gel free (2D)	Untargeted	LC MS/MS
Lété *et al.* [28]	2012	BoHV-4	Gel based + Gel free (2D)	Untargeted	LC MS/MS
Vidick *et al.* [29]	2013	MuHV-4	Gel based	Untargeted	LC MS/MS

CCV: canine coronavirus; KSHV: Kaposi’s sarcoma-associated herpesvirus; MHV6: murine hepatitis virus; HCMV: human cytomegalovirus; EBV: Epstein–Barr virus; MCMV: mouse cytomegalovirus; RRV: Ross River virus; PRV: pseudorabies virus; HSV-1: herpes simplex virus type 1; AIHV1: Alcelaphine herpesvirus 1; CyHV-3: Cyprinid herpesvirus 3; AngHV-1: Anguillid herpesvirus 1; BoHV-4: Bovine herpesvirus 4; MuHV4: Murid herpesvirus 4.

In the targeted analyses, only visually detected protein bands were submitted to enzyme digestion and MS identification. In the untargeted analyses, the entire gel lane is cut into slices (usually 20 to 30 slices), which are then submitted to protein digestion. In this case, gel cutting is made independently of the visual detection of proteins. The rational for choosing the untargeted strategy is that the sensitivity of modern mass spectrometers is higher than the limit of detection of common protein staining protocols. It is thus very common to identify proteins in a gel region where protein staining revealed nothing. However, the untargeted gel-based analysis is somewhat time-consuming because the complete gel needs to be analyzed rather than just the region in which proteins are detected by staining. In contrast, the time needed for both sample preparation and analysis is definitely shorter in the gel-free approaches. This type of a workflow requires minimal sample manipulation and can be much more easily automated. As both the machine time and the manpower are very often limiting factors, the gel free analysis represents a real alternative, especially if several samples need to be analyzed. Nevertheless, for gel-free approaches to be as efficient as gel-based approaches, a 2D-LC workflow is necessary, which requires less common equipment. However, it is not clear how these two strategies compare in terms of efficiency, *i.e.*, number of detected proteins. Indeed, it is highly difficult to compare the efficiency of all of these methods because they have not often been performed in the same lab with the same instrument or started with the same sample. In our lab, we have analyzed four structural proteomes with both 2D-LC MS/MS and SDS-PAGE LC MS/MS [25,27,28] (and unpublished results). Depending on the virus analyzed, one of the two methods was determined to be optimal. The gel free approach proved to be the best for Cyprinid herpesvirus 3 (CyHV-3), whereas the gel-based workflow was more efficient for Anguillid herpesvirus 1 (AngHV-1), Bovine herpesvirus 4 (BoHV-4) and Murid herpesvirus 4 (MuHV-4) [27,28,29].

The only other manuscript that compared gel-based and gel-free approaches was performed on Suid herpesvirus 1 (SuHV-1, also called pseudorabies virus (PRV)) [26]. In this paper, shotgun proteomics (1D-LC MS/MS) was compared to gel-based analysis but in which no chromatographic separation was realized before MS analysis. This study showed a higher efficiency of the gel-free method, but it should be noted that the latter method was not compared to the SDS-PAGE LC MS/MS workflow. An important argument in favor of gel based analysis is the additional information concerning the protein relative migration, which can be very useful for the discrimination of isoforms [28,29]. Nevertheless, it should also be noted that in our lab, the gel-based approach that is only slightly more efficient than the gel-free approaches, double the time needed for the experiment to be conducted. Continuous evolution of mass spectrometer instruments, which are becoming more and more sensitive and fast-running will probably make the use of a complex and time consuming proteins/peptides separation strategy unnecessary in the next few years. Currently, the use of 1D-LC MS/MS on a high resolution-high acquisition rate instrument already allowed us to identify several additional proteins in the CyHV-3 virion sample in a tenth of the time used for previous analyses (unpublished data). However, the improvement in sensitivity raises the question of whether the identified proteins are true structural proteins that are incorporated in the virion or only abundant viral or cellular proteins contaminating the preparation. This question, already noted at the time of the first proteomic analysis of herpesvirus [8], is continually becoming more relevant with instrumental improvement and needs to be addressed through highly efficient sample preparation strategies.

## 3. Sample Preparation and Fractionation

**Figure 2:**
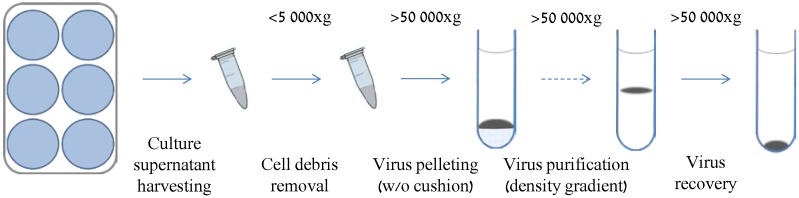
General workflow for the purification of a herpesvirus. Extracellular virions are purified using centrifugation on density gradient, sometimes preceded by concentration on a sucrose cushion. As the purity is of major importance, electron microscopy observation is usually performed to confirm the absence of cell-derived organelles or membrane debris. The proteolysis of partially purified virions is often performed to remove contaminating proteins (dashed line, see also Figure 3).

Determining the protein composition of the viral particles first requires purification of the virions, devoid of as much as possible of cellular contaminants and non-structural viral proteins. This task is usually realized based on differential centrifugation using cushion or density gradients or a combination of both (Figure 2).

To drastically reduce contaminants, herpesviruses are collected from the supernatant of the infected cells at a time when the lysis of the infected cells is still low. In most of the workflows, electron microscopy (EM) is then used to control that cellular organelles or membranes have not been co-purified with viral particles due to a similar density or association with virions. Proteins incorporated in virions are then most commonly identified using SDS-PAGE LC MS/MS or the gel-free procedure. Nevertheless, it is absolutely necessary to validate incorporation of a protein in the viral particle once it has been identified in a proteomic analysis because its presence could be due to contamination by cellular material. A first line of evidence is generally provided by the fact that the protein or some of its homologues have already been proven to be a structural protein. Such an indirect validation presents an important risk of error propagation and proteins defined as structural proteins based only on repeated identification through proteomic analyses should be considered cautiously. In addition, this strategy is poorly effective in virus groups that were not intensely studied through proteomic analysis, such as alloherpesviruses, for which only CyHV-3 and AngHV-1 have been studied.

**Figure 3:**
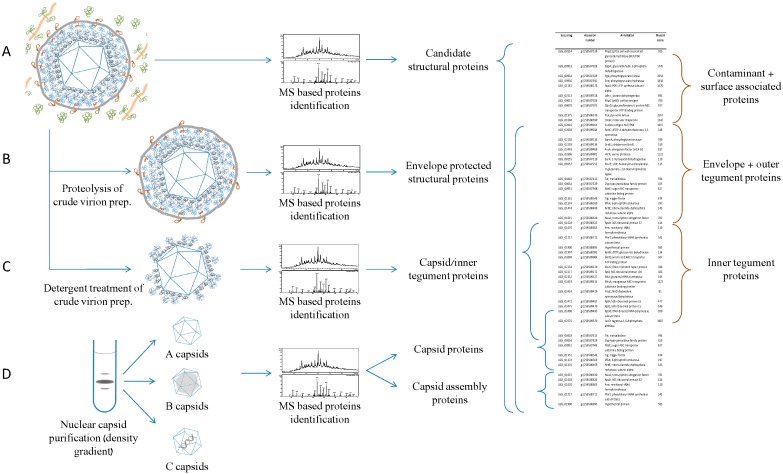
Treatment and fractionation of the virions and nuclear capsids permits accurate determination of the structural proteome. To remove contaminant proteins that could be wrongly identified as structural proteins (**A**), purified virions can be submitted to proteolysis during the isolation procedure (**B**). This procedure permits removal of all of the proteins not protected by the envelope and thus considered as contaminants or surface associated proteins. To determine localization of the proteins inside the virions, detergent treatment to remove the envelope and the so called “outer tegument” proteins has also been shown to be efficient (**C**). Inner tegument proteins are in this case identified in the capsid/tegument fraction. Capsid proteins can generally be predicted based on homology with characterized capsid proteins but can also be identified from purified immature caspids, which help understand the capsid maturation process (**D**). Combination of all or parts of these strategies also allows indirect classification of proteins as “contaminant or surface associated,” “envelope or outer tegument proteins” and “inner tegument proteins” as shown by the red brackets.

The purity can also be validated by the absence of some proteins (or parts of them) known to be nonstructural. Thus, Zhu *et al.* concluded that their KSHV virion preparations were purely based on the absence of glycoprotein B (gB) precursors [19]. A similar strategy was adopted by Loret *et al.* [22], who determined the high level of purity of their virus preparation from the absence of pre-VP22a, VP21 or full length UL26. Another way to confirm the absence of the co-purified organelle is to search for specific markers of this cellular structure. Kramer *et al.* [26], for example, showed that no mitochondrions were co-purified with their extracellular virion by showing that cytochrome C was undetectable in their purified PRV samples. Finally, protease treatment of the virus samples before gradient density purification has been often used [18] to reinforce the robustness of the structural proteome analyses (Figure 3). This type of procedure is supposed to degrade contaminant proteins that present outside of the virus envelope while not affecting proteins present inside the particles because they are protected from proteolysis by the virion envelope. It is important that proteolysis is followed by density purification of the particles to remove the peptides produced and avoid their confusing detection in MS. This approach still allows identification of most of the envelope glycoproteins through the detection of their intracellular and membranous domain [26].

However, the protease treatment could be biased by the proteolysis resistance of some proteins, notably glycoproteins. In our study of MuHV-4 [29], the protease treatment did not affect gB, the envelope glycoprotein detected with the highest number of peptides, and more peptides were even observed for this protein in the proteinase K treated sample than in the control. It should also be mentioned that proteolysis would also be inefficient in eliminating contamination due to the presence of enveloped organelles. Another drawback of this approach is that extracellular virions could be damaged during purification on the sucrose gradient and expose part of the tegument to proteolysis. Finally, proteolysis treatment could also erroneously consider a protein, which is associated with the surface of the virion and that has a role in infectivity, as a contaminant. For example, this is the case for gL, a key component of herpesviruses known to play a role in virus entry [30,31,32,33,34] and stabilized at the surface of the virion only through its interaction with the viral membrane protein gH [35,36]. Thus, while gL was readily identified in untreated PRV and BoHV-4 virions [26,28], it was absent after proteinase K treatment, which could have incorrectly suggested that this protein was a contaminant. The only way to distinguish between contaminants and structural proteins is through immunogold labeling, which allows direct observation by EM of the presence of a protein in association with the virion. Nevertheless, this type of validation is rarely undertaken because it is a highly time-consuming procedure and moreover requires the availability of specific antibodies working in these settings.

As already mentioned, herpesvirus particles are composed of four morphologically recognized structures: the core, the capsid, a dense surrounding layer of proteins (called the tegument), and finally, the envelope composed of a host-derived membrane containing the viral genome encoded glycoproteins. A major goal of virus structural proteomics is to accurately define the composition of these different structures. The capsid composition can be analyzed using a nuclear capsid purification strategy (Figure 3). During virion production, capsid assembly starts through the interaction of the scaffolding protein and a major capsid protein forming the procapsid [37]. After proteolysis of the scaffolding protein, the small capsid protein is recruited, which leads to formation of the intermediate or B capsid that still does not contain DNA. The cleaved scaffolding protein is absent from the C capsid that contains DNA. The third type of stable capsid is called the A capsid and contains neither the scaffolding proteins nor packaged DNA. A, B, and C capsids have different densities and can thus be separated using ultracentrifugation on a density gradient. Nealon *et al.* [13] used this strategy to separately analyze the three capsid types and confirmed that the ORF17.5, the scaffolding protein, was only present in capsid B and that ORF65 is effectively a capsid protein.

Determination of the tegument composition is of major importance in understanding the virion assembly process as recently exemplified by Diefenbach [38]. The analysis of tegument proteins is clearly less straightforward than for the capsid because this structure cannot be purified. Non-ionic detergents can be used to remove the envelope of the virion (Figure 3). Proteins of the tegument are affected differently by this treatment. Indeed, while some of them are highly associated with the capsid, others are removed with the envelope. The produced capsid-tegument sample can be analyzed and a comparison to capsid proteins allows definition of what has sometimes been called the “inner tegument”.

Envelope proteins can normally be identified from the non-ionic detergent obtained samples, but our attempts to use this sample were not successful. Most likely, the amount of envelope proteins in these samples were below the detection threshold. To our knowledge, no specific enrichment of envelope glycoproteins before proteomic analysis has been performed for herpesviruses but it could definitely be a way to improve detection and localization of this important group of proteins. However, the association of some tegument proteins with envelope proteins could also lead to false interpretations [39,40,41,42]. Therefore, proteomic MS analyses can only provide indications regarding the localization of virion proteins. The precise localization of proteins within the viral particle will always require analysis by other approaches such as immunogold labelling and electron microscopy or co-immunoprecipitation.

Surprisingly, we did not detect any peptide corresponding to gN either in MuHV-4 or in BoHV-4 virions. However, as previously mentioned, gN and gM form a complex in herpesviruses [43,44] and gN is needed for the proper processing of gM. Moreover, we readily detected gN by Western blotting on the same MuHV-4 virions preparations even after the proteinase K treatment [29]. The absence of gN in our analyses is therefore likely due to a detection failure by our mass spectrometry approach.

A way to improve proteome coverage is to use multiple enzymes rather than only trypsin, such that regions of the proteome lacking the trypsin cleavage side are also accessible to MS identification. Swaney *et al.* [45] recently compared the results of an extensive proteomic analysis of a complex protein mixture with trypsin alone or with the additional use of four other enzymes. In this study, the number of identified proteins was improved by 20% using the multi-enzyme strategy. This number can be considered relevant and should encourage these types of approaches. The only attempt to apply multiple enzymes to the study of herpesviruses was published by Kunec *et al.* [24], who added moderate proteinase K digestion to the common trypsin strategy. Unfortunately, it is unclear from this publication if proteinase K was only applied to infected cells, in which case proteinase K treatment permits improvement of proteome coverage or also to purified virions. Regardless, no additional proteins are reported in the purified virions due to the use of the additional enzyme. Another interesting aspect of the use of additional enzymes is the improvement in sequence coverage within proteins rather than the increased number of identified proteins. Indeed, Swaney *et al.* [45] showed an important increase in the protein coverage through the use of additional proteolytic enzymes. This improvement could be valuable for detection and discrimination of all types of isoforms in structural proteins (e.g., splicing variant, alternative start/stop codon usage, and post translational processing).

## 4. Post-Translational Modifications of Structural Proteins

The most abundant post-translational modification (PTM) observed in the herpesvirus structural proteins is glycosylation, which affects most of the envelope proteins [46]. Abundant glycosylation can lead to aggregation of glycoproteins into very large complexes, which cannot enter the gel and are thus not detected in SDS-PAGE-based workflows. Moreover, glycosylation can also decrease the efficiency of trypsin digestion by steric hindrance and thus impair protein detection. In addition, glycan moieties are generally highly heterogenic and proteins are present as several glycoforms distributed throughout the gel in the case of the SDS-PAGE-based workflow. This distribution results in a decrease in the effective concentration and thus can negatively influence both the detection and perceived abundance of the protein. Finally, glycosylated peptides are not identified by database search engines because their molecular weight is modified in an unpredictable way. Altogether, these aspects make glycoprotein identification challenging through mass spectrometry. Because virion surface proteins are very often glycosylated, it makes this important protein the most difficult to detect. For this reason, we and others [24] have tested the effect of glycosylation removal on the identification rate of glycoproteins in different herpesviruses. In all of the cases, we observed more peptides for all of the glycoproteins thus suggesting the relevance of the method even if only a few additional proteins were revealed in the deglycosylated samples. Observation of additional peptides through deglycosylation also makes subsequent analyses more efficient such as proteogenomic and protein absolute quantification (see hereafter). When associated with SDS-PAGE-based analysis, protein deglycosylation also allows confirmation of the glycosylation status of a protein through the modification of its relative migration. It should be mentioned that deglycosylation is generally only poorly efficient and should be optimized to significantly improve protein detection.

Multiple structural proteins are also phosphorylated and this modification can have a major effect on viral infectivity [47,48,49,50,51]. For example, ORF47p from varicella-zoster virus, which codes for a kinase similar to CK-2 [52], is responsible for phosphorylation of the tegument protein ORF9p. Riva *et al.* [50] showed that this phosphorylation is important for virion production. Besides the demonstrated importance of phosphorylation of structural proteins in the virus lifecycle, only a few of the structural proteomes of herpesvirus published so far have reported the presence of phosphoproteins. Johannsen *et al.* [16] observed 10 phosphoproteins in Human herpesvirus 4 (HHV-4, also called Epstein-Barr virus (EBV)) most of which were unknown. Phosphorylation not only affected tegument proteins but also capsid, envelope proteins and host proteins. Interestingly, consensus sequences for virion kinase could be identified on some phosphopeptides from both viral and host origin. Kramer *et al.* [26] also found phosphorylation on four viral structural proteins in PRV, pUL26, pUL36, pUL46, and pUL48. Their findings were in contradiction with the precedent findings of Morrison *et al.* [53], which showed that the herpes simplex virus 1 (HSV-1) orthologue of pUL36 was only phosphorylated upon cell entry. This clearly demonstrates the importance of considering phosphorylation as well when analyzing structural proteomes of herpesviruses. Phosphopeptides are usually only poorly represented in regular proteomic datasets and require specific enrichment to be detected in significant numbers [54]. There are three reasons why phosphopeptides are difficult to detect: a) they are present in low amounts, b) they poorly ionize in the ion source and c) they poorly fragment in the mass spectrometer. The detection of multiple phosphorylations in EBV and PRV structural proteomes without any enrichment suggests that this modification is present in large amounts in the extracellular virions and could have unexpected functions in the viral lifecycle. However, while methodologies are well-known and readily applied in several contexts [55], no analysis specifically targeting phosphorylated proteins in structural proteomes of herpesviruses has been reported so far.

Two others PTMs were also frequently described in proteins incorporated in the virion: palmitoylation [56,57,58,59] and myristoylation [59,60]. Although both modifications have been known for a very long time, a dedicated workflow for their selective enrichment and detection in proteomic analyses has only been recently described [61,62] and has appeared less straightforward than in the case of glycosylation or phosphorylation.

## 5. Proteogenomic Analysis

Proteogenomics can be defined as the use of fragmentation spectra acquired in the context of proteomics to improve genome annotation [63,64]. This process can lead to several different improvements of the genomic data quality, the most straightforward one being the confirmation of expression ORFs. Homologies with well characterized sequences can sometime be scarce for divergent groups such as alloherpesviruses. In this case, several ORFs have been regarded as only putative coding sequences because no homology has been found with any known proteins at the time of genome sequencing [17,20,24]. Identification of the proteins coded by these doubtful sequences is a first and important step in the understanding of the virus lifecycle.

Nevertheless, proteogenomics is more often regarded as a way to refine ORF prediction, which is by definition imperfect and sometimes misses coding sequences or inaccurately defines gene boundaries. In particular, small ORFs are probably too often rejected by traditional ORF prediction tools [65]. Thus, proteomic data can be searched against a database construct using translation of all six reading frames of genomic data. Alternatively, a database can be constructed as a stop-to-stop list of ORF that is eventually limited in size. Using such a strategy, a study by Varnum *et al.* [15] highlighted six new short ORFs that were not predicted in the annotated genome of Human herpesvirus 5 (HHV-5, also called human cytomegalovirus (HCMV)). Confidence in their findings comes from the fact that these new ORFs were identified through high confidence hits or with multiple different peptides. Kattenhorn *et al.* [17] similarly identified the product of two previously unannotated ORFs in the Mouse cytomegalovirus (MCMV) structural proteome. For one of the newly detected ORFs, called m166.5 because it is localized between m166 and m167 with partial overlap, the authors confirmed the expression using an HA-tagged m166.5 recombinant virus. In addition to the discovery of two new ORFs, two sequencing errors were detected, which led to extension of the C-terminal end of the m20 gene product and to a frameshift in the second half of M31, which restored full length homology with the RCMV R31 gene. Kunec *et al.* [24] reported the largest set of new ORFs detected through proteogenomic analysis with 17 newly identified coding sequences. The identifications were performed using a highly developed statistical method allowing assignment of significance to individual identifications. All of their new ORFs were detected based on single peptides, which are usually rejected from the proteomic dataset and only seven of them could be assigned a start codon. A total of six out of these seven new ORFs were finally detected at the RNA level using reverse transcription-PCR (RT-PCR) and proving their translation. In contrast with these three examples, in our analyses of MuHV-4 [29] and BoHV-4 [28] structural proteomes, the proteogenomic strategy did not allow us to detect new ORFs. This could be attributed to the exclusion from our analysis of proteins only matching to single peptides and to updates of the genome sequences subsequent to the protein studies.

## 6. Structural Protein Abundance

An important point while determining the structural protein composition is the determination of the absolute abundance of the identified proteins or at least the ability to rank them. Therefore, mass spectrometry-based protein identification cannot be considered a quantitative method [66]. Different factors can influence peptide detection in the mass spectrum and this parameter is not directly usable for absolute quantification. Nevertheless, indirect measurements can easily provide semi-quantitative data such as spectral counting [67]. Spectral counting is based on the basic assumption that the more abundant a protein is in a sample, the more often one of its peptides will be selected for fragmentation by the mass spectrometer. Spectral counting accuracy has been shown to depend on the number of detected spectra [68]. With this as the basic principle, numerous different indexes have been developed and comparisons of some of them are available [69]. In our four studies on the structural proteomes of herpesviruses, we used the exponentially modified protein abundance index (emPAI) [70]. This measure takes into account the number of identified peptides and the number of theoretical detectable peptides. The data obtained through the use of this index appeared realistic because known major proteins, such as major capsid proteins obtained systematically, were one of the highest scores observed. Nevertheless, by comparing their abundance data (that was obtained through the NQPCT score [71] (number of unique peptides)) to the published molar ratio of viral components, Loret *et al.* found that the correlation was rather low (never exceeding 0.42). This clearly demonstrates that the protein abundance determined using spectral counts should only be considered semi-quantitative data.

## 7. Host Proteins

Host proteins have been reported to be present in all structural proteomes for which they have been searched. The presence of such proteins associated with or incorporated in virions raises several questions. First, are these proteins effectively associated with the particle or do they represent contamination of the purified virions? As for virus encoded proteins, a first level answer to this question is the degree of purity of the virions preparation, which needs to be as high as possible. Proteolysis of the particle preparation has also been frequently used as a means to demonstrate incorporation of cellular proteins into the virion [16,26,28,29]. As previously mentioned, while generally accepted as a good validation of particle incorporation, this procedure still suffers from some limitation. For instance, some proteins could be, at least partially, intrinsically resistant to proteolysis due to examples from PTMs, conformation or polymerization. In our study of the MuHV-4 and BoHV-4 structural proteome, we observed a decrease in the number of identified host proteins in samples treated with proteinase K in comparison with control samples [28,29]. In addition, the relative abundance of most of the host proteins was decreased after protease treatment, which would suggest that at least part of the detected host proteins are outside the viral particle and represent potential contamination. However, as has been proposed by Kramer *et al.* [26], proteins associated with the external surface of virions could be of major importance for the virus lifecycle but would not be resistant to proteolysis and considered as contaminant proteins using a proteolysis-based validation strategy. Some host proteins are quite often detected in virions such as actin tubulin, glyceraldehyde 3-phosphate dehydrogenase (GAPDH), HSP70/HSC70, and annexin (Table 2) and the presence of some of them inside the particle has been validated or suggested using alternative approaches. For example, this is the case for actin, which has been found in almost all analyzed herpesvirus structural proteomes and for which alternative methods have also suggested incorporation with the virions [72,73]. Other proteins from the cytoskeleton are very often identified such as Ezrin-radixin-moesin [18,29,61,72]. Ezrin, which is a cross-linker between the actin cytoskeleton and plasma membrane, has been attributed to a function in an early stage of the infection of Human herpesvirus 8 (HHV-8, also called Kaposi sarcoma associated herpesvirus (KSHV)) [74]. The role of these host proteins present in virions is still relatively unclear and represents another important question. Probably some of these host proteins serve in transport, assembly, and egress of the particle but others could have functions in virus entry. Annexin A2 is a phospholipid binding protein that functions as a membrane recruiter for several proteins [75]. The role of annexin A2 in herpesvirus infection is controversial with some studies reporting a positive effect on virus entry [76,77] in HCMV, whereas other studies reported no influence [78]. In MuHV-4, we did not observe any difference in terms of the growth rate on Annexin A2 deficient mouse embryonic fibroblasts. Nevertheless, we could not exclude that cell type has an important influence on the host protein effect on infectivity as has been suggested in human immunodeficiency virus type 1 *(*HIV-1), which also incorporates annexin A2 [79]. To our knowledge, the influence of cell type on the composition of the incorporated host proteins has never been studied but could give new insights into their functions. The repeated presence of highly abundant cellular proteins, such as GAPDH, for which no obvious function can be proposed also raises a third question regarding the specificity of the incorporation of the host proteins. Specific incorporation should be understood as a result of an enrichment process, which makes the protein proportionally more abundant in the particle than in the host cell. To date, no dedicated workflow has been proposed to answer this question and only intracellular abundance of the host protein can give clues to its specific incorporation or not in virions. Further studies are required to define more precisely the level and importance of cellular proteins in herpesvirus virions.

## 8. Mutual Influence of the Structural Proteins/Quantitative Analyses

Analysis of the targeted mutant is a frequent strategy to decipher gene functionality. However, when dealing with structural proteins, it is of major importance to ensure that the effect observed upon deletion of a gene is due to the lack of the encoded protein and not due to the impaired incorporation of other viral or even cellular proteins. For example, in PRV, it has been shown that UL20 deletion impacted gK processing and incorporation [80]. In Human herpesvirus 2 (HHV-2, also called Herpes simplex virus 2 (HSV-2)), Matsuzaki *et al.* showed that deletion of US3 impaired incorporation of UL46. Even host protein incorporation can be affected; del Rio *et al.* [81] showed that actin was more abundant in PRV virion in the UL49 deletion mutant. Overall, these observations were conducted by Mettenleiter’s group who analyzed the structural proteomes of PRV mutants lacking several tegument proteins [21,82,83]. These analyses were based on a SILAC (stable isotope labeling by amino acids in cell culture) approach in which the relative abundance of proteins can be accurately measured based on metabolic labelling during cell culture [84]. This very powerful workflow allowed Michael *et al.* [83] to determine that the UL47 deletion also influences actin incorporation virions to a level even higher than observed for the UL49 deletion. This method even allowed determination that the UL47 and UL49 deletion also had an effect on the isoform of pUL48 that was incorporated in the particles. Deletion of UL11 and UL16 also modified the isoform of pUL36 which is incorporated with a specific accumulation of the N-terminal fragment in the mutants [56]. Finally, the influence of the presence of pUL21 on incorporation of pUL46, pUL49, and pUS3 was also determined using the same approach and enabled the explanation of the attenuated phenotype of the PRV Bartha strain [82]. The very fruitful application of quantitative proteomics to structural proteomes of herpesviruses performed by Mettenleiter’s group should encourage this type of research. Many biological questions would benefit from such experiments, not only regarding the mutual influence of the incorporation of structural proteins, but also in the context of the influence of the cell type on the production of virions and their infectivity. Incorporation and the role of host proteins could also be better understood through quantitative proteomic analysis.

**Table 2:** Host proteins frequently detected associated with the virions.

	*Davison et Davison*	*Bortz et al.*	*Johannsen et al.*	*Varnum et al.*	*Kattenhorn et al.*	*Zhu et al.*	*Bechtel et al.*	*Michael et al.*	*Loret et al.*	*Dry et al.*	*Michel et al.*	*Kramer et al.*	*Van Beurden et al.*	*Lété et al.*	*Vidick et al.*	
*Year of publication*	*1995*	*2003*	*2004*	*2004*	*2004*	*2005*	*2005*	*2006*	*2008*	*2008*	*2010*	*2011*	*2011*	*2012*	*2013*	
*Virus*	*CCV*	*MHV68*	*EBV*	*HCMV*	*MCMV*	*KSHV*	*KSHV*	*PRV*	*HSV-1*	*AlHV1*	*CyHV3*	*PRV*	*AngHV*	*BoHV4*	*MuHV4*	
***Proteolysis***	(-)	(-)	(-)	(-)	(-)	(-)	(+)	(-)	(-)	(-)	(-)	(+)	(-)	(+)	(+)	
***Number of identified host proteins***	1	5	6	71	7	21	9	4	49	6	18	48	28	15	31	**Total occurrence**
Actin (a)	+	+	+	+	+	+	+	+	+	+	+		+	+		13
Annexin A2, A1, A3 (a)		+		+	+	+		+	+	+	+	+		+	+	11
Cofilin				+	+				+		+	+		+	+	7
Elongation factors (a)				+	+	+	+				+			+		6
Heat shock protein 70				+		+	+	+	+				+			6
14-3-3 (a)				+		+					+	+		+		5
Glyceraldehyde-3-phosphate dehydrogenase				+	+				+				+		+	5
Heat shock protein 90			+	+		+	+						+			5
Rab (a)						+			+		+	+			+	5
ADP ribosylation factors (a)				+					+		+	+			+	5
Caseine kinase				+					+		+	+			+	5
Histones (a)					+					+					+	3
Pyruvate kinase						+	+			+						3
Profilin									+				+	+		3
Tubulin (a)				+			+				+					3
Rab GDP dissociation inhibitor beta-like				+	+										+	3
S/T-protein Pase PP1-alpha				+								+			+	3
Moesin				+			+								+	3
cyclophilin A1				+		+			+							3
Enolase				+		+	+									3

## 9. Conclusions

Proteomic analysis of structural proteins definitely contributes to the understanding of the herpesvirus lifecycle. Sample preparation is of major importance in this task because it is always difficult to assess if a protein is effectively incorporated in the virion or just represents a contaminant. Analysis of PTMs on structural proteins, which seems to be more common than was previously thought, could also be highly valuable in the better understanding of cell targeting and the infectivity of the particles. Studies using mutants also revealed important mutual influences between some structural proteins. This aspect would currently benefit from the development of more quantitative analyses in which this influence could be characterized in more detail and on more than a few strains. It is also clear that some host proteins are specifically incorporated in the virion, raising the question of the role of these proteins in infectivity or in particle production. Again, the ability to quantify structural proteomics would increase our understanding of these phenomena.
